# Oxygen regulation in the earliest animals: spatiotemporal resolution of oxygen dynamics in single-osculum *Halichondria panicea* and *Haliclona rosea* sponges

**DOI:** 10.3389/fphys.2026.1849630

**Published:** 2026-07-29

**Authors:** Lars Kumala, Malte Thomsen, Donald E. Canfield

**Affiliations:** 1 Nordcee, Department of Biology, University of Southern Denmark, Odense, Denmark; 2 Marine Biological Research Centre, University of Southern Denmark, Kerteminde, Denmark; 3 Danish Institute of Advanced Study (DIAS), University of Southern Denmark, Odense, Denmark

**Keywords:** anoxia, contractile behavior, early animals, oxygen, oxygen oscillations, planar optode, respiration, sponge explant

## Abstract

Sponges regulate internal water flow and oxygen distribution through contractile behavior, yet the mechanisms governing spatio-temporal oxygen dynamics remain poorly understood. Resolving these dynamics in sponges is crucial for understanding sponge host-microbiome interactions, tissue homeostasis and growth. We applied 2-D luminescence lifetime imaging of oxygen to unveil temporal as well as spatial oxygen dynamics in single-osculum explants of two demosponges: *Halichondria panicea* and *Haliclona rosea*. Our study reveals intrinsic deoxygenation in explants during episodes of osculum contraction generating an oxygen gradient with increasing concentrations towards the explant periphery. Furthermore, time-lapse oxygen imaging uncovered contrasting spatio-temporal patterns of internal O_2_ distribution in the examined individuals of the two species. Four explants of *H. panicea* faced periodic and consistent episodes of internal oxygen drawdowns and centralized anoxia, followed by reoxygenation. Periods of internal anoxia persisted for up to 4.5 h of the total 92.1-hour observation period. Conversely, two *H. rosea* explants displayed versatile deoxygenation patterns, including recurring, propagating waves of oxygen reduction, with relatively few areas experiencing anoxia over the course of the 70.6-hour observation period. The observed oxygen dynamics indicate that filter-feeding sponges may employ distinct behavior to actively regulate their internal oxygenation. We suggest that spatiotemporal oxygen regulation may represent an ancestral trait that emerged in early diverging metazoans, and possibly in response to increasing levels of atmospheric oxygen.

## 1 Introduction

Oxygen regulation is fundamental for maintaining internal homeostasis and for facilitating essential biological processes in animals, such as cellular respiration, tissue regeneration and the mitigation of oxidative stress (Jagannathan et al., 2016; Hammarlund et al., 2018; Lee et al., 2022). Many animals, such as mammals or termites, establish oxygen gradients that shape the spatial arrangement and composition of aerobic and anaerobic microbial communities within their bodies (Brune et al., 1995; Albenberg et al., 2014). A prominent example is the human gut, where a steep oxygen gradient from the mucosal surface towards the lumen sustains symbiotic microbes essential for nutrient cycling and digestion (Espey, 2013; Albenberg et al., 2014; Singhal and Shah, 2020).

Beyond metabolism, oxygen and its reactive oxygen species (ROS) function as signaling molecules that regulate processes such as cell differentiation, wound healing and tissue regeneration (Rodriguez et al., 2008; Bartz and Piantadosi, 2010; Abdollahi et al., 2011; Hammarlund et al., 2018). In most animals, hypoxia stimulates angiogenesis and cellular repair via the hypoxia-inducible factor (HIF) pathway (Semenza, 2000; Mills et al., 2018; Hammarlund et al., 2020b). Additionally, temporal fluctuations in oxygen levels, including periodic hypoxia, may contribute to cellular resilience by limiting ROS-induced damage (Droge, 2002; Mateika et al., 2015). These mechanisms highlight the importance of both spatial and temporal oxygen regulation in shaping physiological adaptations across animal taxa.

The evolution of specialized organs and compartmentalization in animals marked a pivotal advancement in oxygen regulation. As body plans evolved, increasing size and complexity necessitated specialized structures for oxygen uptake and transport (Fenchel and Finlay, 1995; Schmidt-Nielsen, 1997). Specialized structures such as gills and lungs efficiently extract oxygen from the environment, while circulatory systems distribute it to metabolically demanding tissues (Schmidt-Nielsen, 1997). Compartmentalized body plans facilitated the maintenance of internal low-oxygen niches, supporting hypoxia-driven processes like tissue regeneration and oxygen-sensitive microbial symbioses (Nyholm and Graf, 2012; Semenza, 2000). However, it remains unclear how early animals with simple morphologies and no specialized respiratory organs or distinct body compartmentalization regulated the distribution of internal oxygen.

Filter-feeding sponges (*Porifera*) offer a key model system for studying oxygen regulation in early branching metazoans with primitive body plans. Together with ctenophores, sponges occupy one of the most contentious positions in animal phylogeny, and conflicting evidence supports either *Porifera* (e.g. Redmond and McLysaght, 2021) or *Ctenophora* as the earliest-diverging metazoan lineage (e.g. Schultz et al., 2023). Yet, their circulatory system represents one of the most basal forms of internal transport, offering insights into the fundamental mechanisms of oxygen distribution before the evolution of specialized respiratory structures. Unlike more advanced animals, sponges lack a centralized “pump” to transport oxygen, such as a heart. Instead, active water-pumping is accomplished by the coordinated beating of flagellae in specialized cells known as choanocytes, a cell type unique to sponges. These choanocytes are organized into choanocyte chambers, which drive unidirectional water flow through an internal aquiferous system composed of inhalant and exhalant canals (Kilian, 1952; Fjerdingstad, 1961; Larsen and Riisgåd, 1994; Leys et al., 2011). This water flow supplies the sponge interior with oxygen, food particles and dissolved compounds that can serve as substrates for various metabolic pathways (e.g. Hudspith et al., 2021). Oxygen transport through the complex aquiferous system promotes oxygen diffusion into interior sponge cells, in addition to diffusive flux across the sponge’s outer surface, the exopinacoderm (Kumala and Canfield, 2018). Understanding how these simple organisms modulate internal oxygen levels provides valuable insights into the evolutionary foundations of oxygen regulation in the animal kingdom.

Despite lacking muscles and a nervous system, sponges actively regulate the intensity of internal water flow by contracting and expanding their body walls. The contraction-expansion behavior is mediated by pinacocytes that line internal body cavities and canals (Nickel et al., 2011; Musser et al., 2021; Colgren and Nichols, 2022), and involves the relaxation and re-establishment of tension in actomyosin stress fibers within this pinacoderm tissue (Ruperti et al., 2024). Sponges show distinct behavioral patterns in response to internal as well as external stimuli. The behaviors range from osculum closure and localized, propagating, spasms to coordinated, whole-body deflations that expel particulate matter (Elliott and Leys, 2007; Nickel et al., 2011; Kornder et al., 2022; Harrison et al., 2024; Ruperti et al., 2024). These behaviors can be stimulated by elevated food and sediment loads (Strehlow et al., 2017; Pineda et al., 2017; Kumala and Riisgård, 2024), changes in ambient water flow (Riisgård et al., 2016), and electrical, mechanical and chemical cues (Parker, 1910; Prosser et al., 1962; Elliott and Leys, 2007).

Sponge contractions include the temporal constriction of water canals, ostia, and/or oscula, which modulates internal water flow without directly affecting flagellar activity of choanocytes (Leys et al., 2024). This behavioral control of aquiferous system dimensions induces pronounced reductions, and in some cases cessation, of water flow and hence periods of internal anoxia (Hoffmann et al., 2008; Kumala and Canfield, 2018). These transient oxygen fluctuations influence the sponge’s internal redox state and likely the activity of its associated microbial consortia (Taylor et al., 2007; Hoffmann et al., 2009; Kumala et al., 2021). This spatial and temporal oxygen depletion may facilitate nutrient cycling via coupled aerobic and anaerobic processes within the sponge holobiont (Hoffmann et al., 2009; Schläppy et al., 2010a). However, the relationship between sponge contractions and the spatiotemporal organization of subsequent internal deoxygenation remains poorly resolved.

The aquiferous system in sponges, such as in *Halichondria panicea* and *Haliclona rosea*, is subdivided into aquiferous modules, each supplying a distinct volume of the sponge. Each module consists of a network of inhalant pores on the sponge surface, choanocyte chambers and water canals, all converging into a single exhalant opening known as an osculum (Fry, 1979; Ereskovskii, 2003). In large sponges with multiple oscula, asynchronous contractions of individual modules may allow for localized control of oxygen distribution, maintaining spatial heterogeneity to fulfil varying metabolic demands (cf. Reiswig, 1971; Schläppy et al., 2010b; Kumala et al., 2021). However, it remains unknown whether heterogeneous oxygenation including anoxia is a universal feature of sponges, including those with only one aquiferous module.

To investigate species-specific patterns of oxygen regulation in sponges, we grew single-osculum sponges on transparent oxygen-sensitive planar optodes. When combined with luminescence lifetime imaging (Glud et al., 1996; Holst et al., 1998), we obtained time-resolved 2-D imaging of internal sponge oxygen levels. These techniques have been instrumental in studying oxygen dynamics in basal metazoans, including corals (Kühl et al., 2008), and have also been applied previously to *H. panicea* (Kumala et al., 2021). However, other than *H. panicea*, relatively little is known about the spatiotemporal dynamics of internal oxygen fluctuations in other sponge species, although the occurrence of internal anoxia has previously been documented (Hoffmann et al., 2005a, Hoffmann et al., 2005b, Hoffmann et al., 2008; Schläppy et al., 2010b). Here, we reanalyzed the *H. panicea* dataset using a spatially resolved analytical framework that extends previous temporal event-based analyses of deoxygenation dynamics. This framework quantifies oxygen distributions at the pixel level, spatial heterogeneity, and space-time patterns of deoxygenation. The same analytical pipeline was then applied to *H. rosea* for comparative analyses of oxygen dynamics between two demosponge species. Our findings reveal contrasting patterns of internal oxygen dynamics in single-osculum *H. panicea* and *H. rosea* sponges. These results suggest that spatio-temporal regulation of internal oxygen may be an ancestral trait that predates the evolution of more complex body plans in metazoans with their own internal oxygen regulation.

## 2 Methods

We applied modular luminescence lifetime imaging (Holst et al., 1998; Holst and Grunwald, 2001; Kumala et al., 2021) to investigate temporal and spatial changes in oxygen distribution in single-osculum *Haliclona rosea* sponge explants. To ensure comparability between species, the experimental setup and oxygen imaging methodology were identical to those previously established for single-osculum *Halichondria panicea* explants (Kumala et al., 2021), utilizing planar optodes to visualize oxygen dynamics in the sponge interior. Planar optodes operate through dynamic quenching of luminescence by oxygen (O_2_), using an immobilized indicator dye (DeGraff and Demas, 2005). To minimize methodological variation, both datasets were generated using the same sensor cocktail batch containing both indicator and antenna dyes. Optode preparation, lifetime imaging procedures, and calibration protocols followed identical workflows as described in Kumala et al. (2021). The maximum theoretical spatial resolution of the imaging system was ~100 µm × 100 µm.

### 2.1 Preparation and cultivation of sponge explants

Using the methods outlined in Kumala et al. (2017), Kumala et al, 2021), single-osculum explants of the demosponge *H. rosea* were obtained by excising fragments (~100 mm³) from parent specimens collected in Kerteminde Fjord on the island of Fyn in Denmark. This approach generated explants of comparable overall shape and thickness, facilitating comparative analyses between the two sponge species. As in previous experiments on *H. panicea*, the sponge cuttings were placed on the planar optodes after submerging the flume in a flow-through aquarium (30 L) containing well-aerated, bio-filtered (*Mytilus edulis*) seawater maintained at ~15 °C and a naturally fluctuating salinity of 18–22. Within 3–6 days, these cuttings attached to the optode surface and developed an osculum over the following 5–12 days, signaling the restoration of their aquiferous system and the resumption of active filter-feeding. These single-osculum explants were regularly (i.e. 1–2 times day^-1^) fed with lab-cultured *Rhodomonas salina* algal cells (~5000 cells mL^-1^) prior to and during the experimental period. Only actively pumping explants (*n* = 3), confirmed through the uptake and expulsion of a fluorescent dye, were included in the experiments. However, one explant was excluded from the analyses because parts of the sponge had grown above the seawater surface during cultivation, potentially affecting its behavior and oxygen dynamics. Thus, only two fully submerged *H. rosea* explants were included in the image analyses.

### 2.2 Experimental setup

As for our earlier work with *Halichondria panicea* (Kumala et al., 2021), experiments were conducted in fully oxygenated, 0.2-µm filtered (initially) seawater (salinity of 20) supplemented twice daily with *Rhodomonas salina* algae. Recirculation and water flow was maintained by an aquarium pump directing outflow into a 2L beaker beneath the flume’s exit. Temperature was held constant at 15.1 ± 0.2 °C using a temperature controller. All experiments were performed in darkness to prevent photosynthetic activity by sponge-associated microorganisms from affecting O_2_ measurements and masking intrinsic oxygen fluctuations associated with sponge ventilation behavior. For calibration of O_2_ images, the oxygen concentration was regulated with a gas mixer (Brooks Instrument, Model 0154) and monitored using a FireStingO_2_ optical oxygen meter (Pyro Science, Germany) connected to a computer with Pyro Oxygen Logger® software. Oxygen readings were corrected for changes in temperature by the software using an external temperature sensor placed in the flume water.

### 2.3 Image and data analysis

Bottom-view time-lapse images of the explant base were analyzed in ImageJ (Version 1.47g). For each specimen, we manually determined a region of interest (*ROI*) including the area of the explant base (*A*, mm^2^) grown on the planar optodes as observed in bottom-view images and where pixel counts were converted into mm^2^ using a reference scale bar. Image sequences were transformed to a 32-bit greyscale format to convert each pixel’s brightness/luminescence signal into an oxygen measurement using the calibration method outlined in Kumala et al. (2021). The O_2_ concentration at the explant base of all explants was determined for each image of the image sequence using the ‘multiple measure’ function in the ROI manager in ImageJ.

The same oxygen threshold analyses were applied to oxygen images from both sponge species. For *H. rosea* (*n* = 2) and *H. panicea* (*n* = 4), we adjusted the maximum threshold values to determine the (cumulative) relative area of the sponge base experiencing various ranges of oxygen levels, i.e. < 70%, < 60%, < 50%, < 40%, < 30%, < 25%, < 20%, < 15%, < 10%, < 5% and < 1% air saturation. These threshold values were used as consistent criteria to determine the frequency and magnitude of deoxygenation events across both datasets. Anoxia was defined as oxygen levels below 1% air saturation (< 1% AS). Ambient O_2_ levels were derived from the mean O_2_ measurements of three selected areas (each containing 25 × 25 pixels) located outside of the sponge.

To resolve spatial and temporal O_2_ dynamics within the explant base, data analyses were performed in R (version 4.1.1, R Core Team, 2015). We determined the proportion of pixels within the explant exhibiting O_2_ levels below 10%, 4%, and 1% air saturation at least once during the entire experiment, independent of the timing of deoxygenation events. To identify spatial hotspots of recurrent anoxia within the sponge base area, we calculated the relative frequency with which each pixel experienced O_2_ levels below 1% air saturation throughout the experiment. These frequencies were visualized using a linear grayscale, with darker shades representing increasingly frequent anoxic events. The pixel with the highest frequency of anoxia was assigned black, and all remaining pixels were scaled relative to this maximum using 256 gray levels.

To investigate the synchronization of oxygen fluctuations across different regions of the sponge, we measured the mean O_2_ level in segmented areas at the base of sponge explants. For this, we developed a macro in ImageJ to partition the *ROI* encompassing the base area*, A*, into equally sized squares. This enabled time-series plots of the mean O_2_ levels for each segment.

We studied the emergence and development of spatiotemporal patterns in oxygen dynamics using space-time plots, with time (hours) on the x-axis and spatial position (pixel distance) on the y-axis. Oxygen saturation was measured in (~100 × 100 μm) pixels along a vertical transect line crossing the explant base. Air saturation was visualized in 256 gray levels, ranging from white (100% air saturation) to black (< 1% air saturation), with pixel positions mapped along the transect line.

## 3 Results

### 3.1 Deoxygenation dynamics

**Figure 1:**
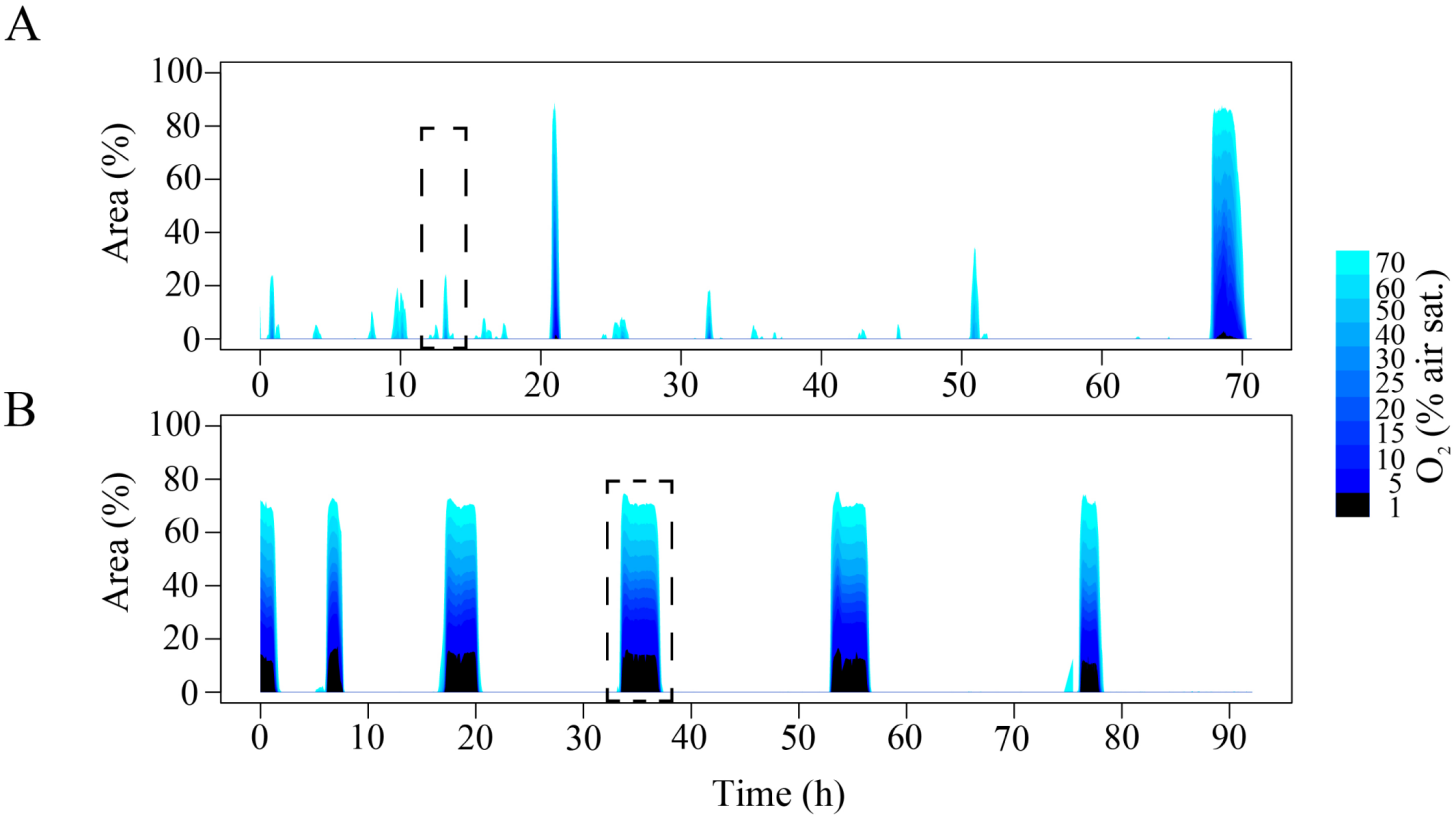
Temporal dynamics of internal oxygenation in single-osculum demosponge explants. Stacked area plots display the cumulative relative area of the explant base experiencing different oxygen saturation ranges over time for representative individuals of **(A)** *H. rosea* (*ID*2) and **(B)** *H. panicea* (*ID*3). Oxygen levels (% air saturation) are color-coded according to the accompanying scale, illustrating transient deoxygenation events in the sponge interior. Dashed boxes highlight specific deoxygenation events that are shown in greater detail by time-lapse imaging in **Figure 2**. Corresponding temporal plots for all other examined individuals are provided in **Supplementary Figure 1**.

Oxygen levels measured at the base of single-osculum *Haliclona rosea* (*n* = 2, *ID*1-2) and *Halichondria panicea* (*n* = 4, *ID*1-4) explants periodically approached those of the ambient seawater during the 70.6-hour and 92.1-hour observation periods (**Figure 1** & **Supplementary Figure 1**). However, both sponges exhibited distinct deoxygenation events at both irregular (*H. rosea*) and, in some cases, regular (*H. panicea*) intervals, during which internal O_2_ levels measured within the base area dropped below 70% air saturation (AS). These events accounted for 28.1% (*ID*1) and 24.5% (*ID*2) of the total observation period in the two *H. rosea* explants. In the four *H. panicea* explants, deoxygenation events ranged from 20.9% (*ID*3) to 31.1% (*ID*2) of the total observation period, with a mean (± 95% confidence interval, CI) of 26.8% ± 7.6% (**Supplementary Table 1**). We recorded 20 (*ID*1) and 28 (*ID*2) deoxygenation events in the *H. rosea* sponges, lasting an average (± 95% CI) of 60 ± 17 min in *ID*1 and 37 ± 13 min in *ID*2. In *H. panicea*, the number of deoxygenation events varied from 5 (*ID*1) to 11 (*ID*4), whereas mean event durations (± 95% CI) ranged from 125 ± 73 min (*ID*4) to 335 ± 155 min (*ID*1) across individuals.

**Figure 2:**
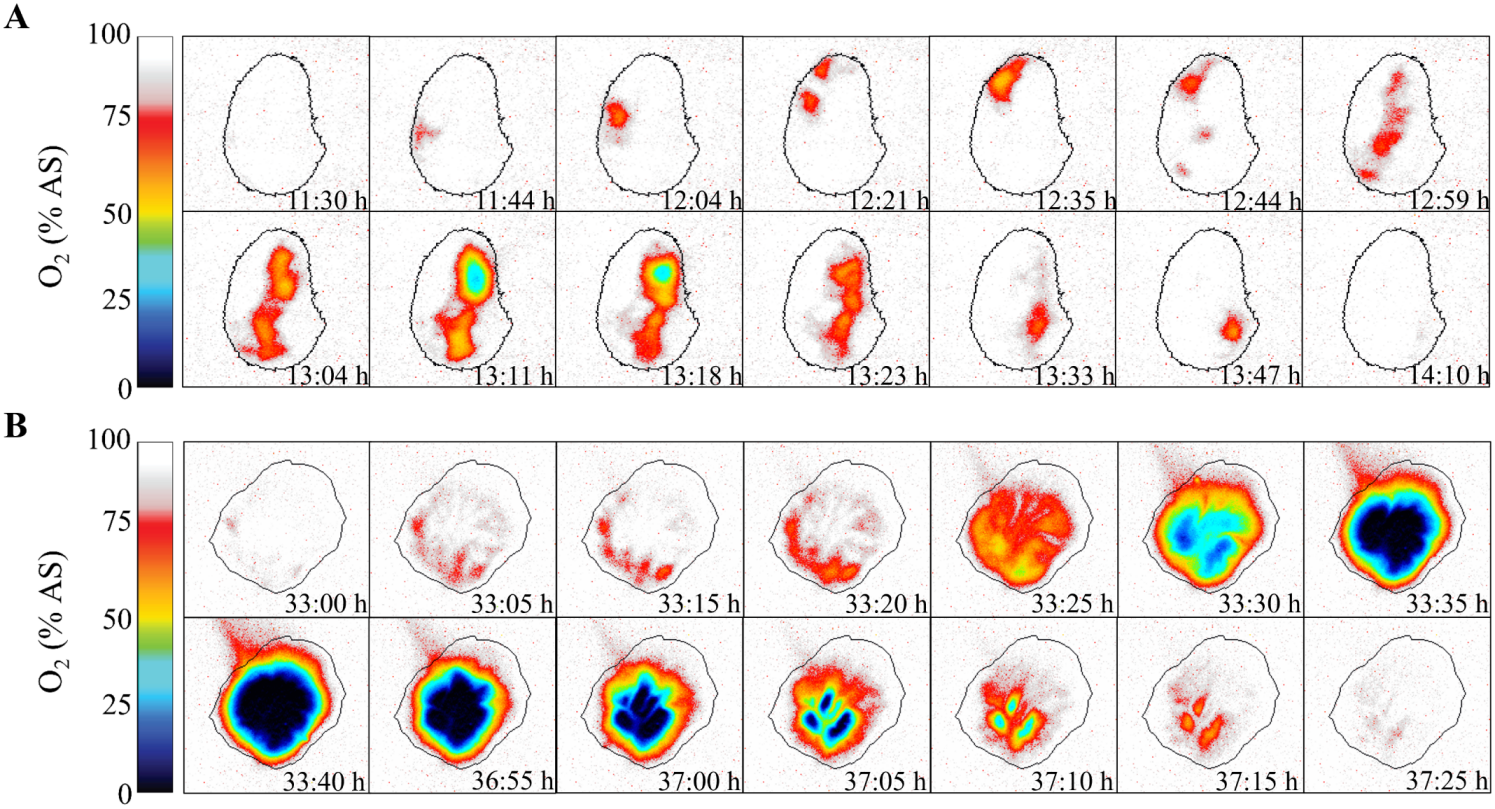
Time-lapse images from two events depict changes in oxygen levels in two demosponge species. Oxygen distribution is shown at the base of **(A)** *H. rosea* (*ID*2) and **(B)** *H. panicea* (*ID*3) sponge explants in fully oxygenated (100% air saturation) ambient seawater (salinity of 20) with added *R. salina* algal cells. Air saturation levels are represented by color. The black line indicates the contour area (i.e., base) of the sponge grown on the oxygen-sensitive planar optode.

We observed substantial differences in oxygen dynamics between the two species (**Figures 1**, **2**; **Supplementary Figure 1**, **Supplementary Table 1**). *H. rosea* explants exhibited sporadic episodes of internal deoxygenation that varied in extent and duration. In *H. rosea* sponge *ID*2, for instance, we recorded 28 distinct deoxygenation events, with oxygen most frequently (i.e. 19 of 28 times) reaching levels between 70 to 40% AS (**Figure 1A**; **Supplementary Table 1**). Similarly, in *H. rosea ID*1, oxygen levels reached 70 to 40% AS during 10 of the 20 recorded deoxygenation events (**Supplementary Figure 1**; **Supplementary Table 1**). While most deoxygenation events in *H. rosea* were brief and moderate in intensity, both individuals occasionally exhibited more pronounced oxygen reductions, including transient declines below 5% AS, lasting up to 109 min. In contrast, *H. panicea* explants repeatedly experienced prolonged and consistent internal deoxygenation, including anoxic periods (< 1% AS) lasting up to 270 min (**Figure 1**; **Supplementary Table 1**).

We noticed complete anoxia in only one *H. rosea* specimen (*ID*2) during two (of the 28) deoxygenation events, resembling patterns recorded in *H. panicea* (**Figures 1A, B**, **2**). During these events in *H. rosea* (*ID*2), the anoxic area (defined as < 1% air saturation) covered, on average (± 95% CI), 1.0% ± 0.1% of the total sponge base over time, with a maximum instantaneous extent of 3%. By comparison, anoxia in *H. panicea* (*ID*1-4) covered an average (± 95% CI) of 8.0% ± 5.5% of the sponge base area, reaching up to 20.9% in specimen *ID*4 (**Figure 1**).

**Figure 2** provides representative examples of deoxygenation events in both species. In *H. rosea*, the event shown lasted approximately 2 hours and originated near the sponge base periphery (**Figure 2A**). Only a relatively small fraction of the sponge experienced changes in oxygen levels at a time, and no areas approached anoxia. Conversely, *H. panicea* exhibited a centralized anoxic zone that persisted for several hours, forming a distinct oxygen gradient with increasing concentrations towards the explant periphery (**Figure 2B**).

### 3.2 Spatial O_2_ dynamics

**Figure 3:**
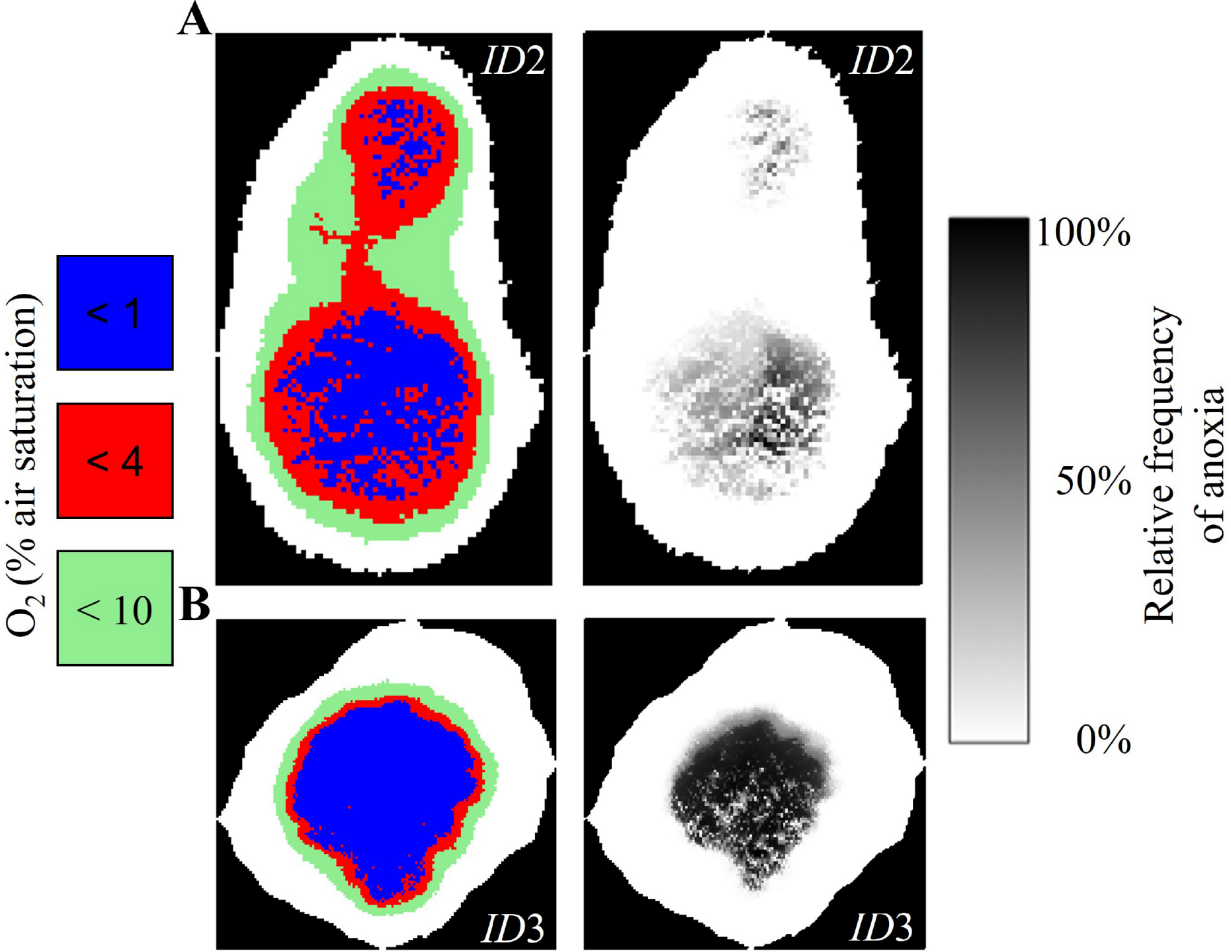
Spatial distribution and frequency of internal hypoxia and anoxia in single-osculum demosponge explants. The spatial extent of deoxygenation is shown for representative individuals of **(A)** *H. rosea* (*ID*2) and **(B)** *H. panicea* (*ID*3). Left column: Colored regions delineate the cumulative relative area of the explant base that experienced specific oxygen depletion thresholds (< 10% [green], < 4% [red], and < 1% [blue] air saturation) at least once during the respective observation period. Pixels outside the sponge base are masked in black. Right column: Grayscale maps illustrate the relative frequency of anoxia (< 1% air saturation) in each pixel. Darker shades indicate a higher frequency of anoxic events. The darkest pixel (solid black) represents the maximum anoxia frequency, occurring in 101 of 4241 measurements (~2.4%) for *H. rosea* and 231 of 5526 measurements (~4.2%) for *H. panicea*.

Our spatial pattern analyses revealed that over time hypoxia (O_2_ < 10% air saturation) affected a substantial portion of the sponge base in both demosponge species (**Figure 3**; **Supplementary Figure 2**). However, we also noticed differences between the two sponge species, as well as between the two *H. rosea* specimens. For instance, only in *H. rosea* explant *ID*2 did we identify distinct areas (two in total) that experienced anoxia (O_2_ < 1% air saturation) at least once during the entire observation period, whereas no anoxic regions were detected in *H. rosea* explant *ID*1. This pattern contrasts with *H. panicea* explants, where deoxygenation, including anoxia, consistently occurred in a centralized manner in all specimens.

Frequency analyses revealed that in *H. rosea* (*ID*2), the pixel experiencing the highest frequency of anoxia was anoxic for 101 (= 1.7 h) out of 4241 measurements over the 70.6-hour observation period (**Figure 3A**). Meanwhile, *H. panicea* explants displayed a larger spatial extent of anoxic areas, with the most affected pixel experiencing anoxia in 231 out of 5526 measurements (*ID*3, **Figure 3B**), amounting to 13.1 hours over the 92.1 hour observation period. These findings underline the more extensive and persistent occurrence of anoxia in the examined *H. panicea* explants compared to *H. rosea*, emphasizing pronounced differences in oxygen dynamics at the sponge base during the observation period.

**Figure 4:**
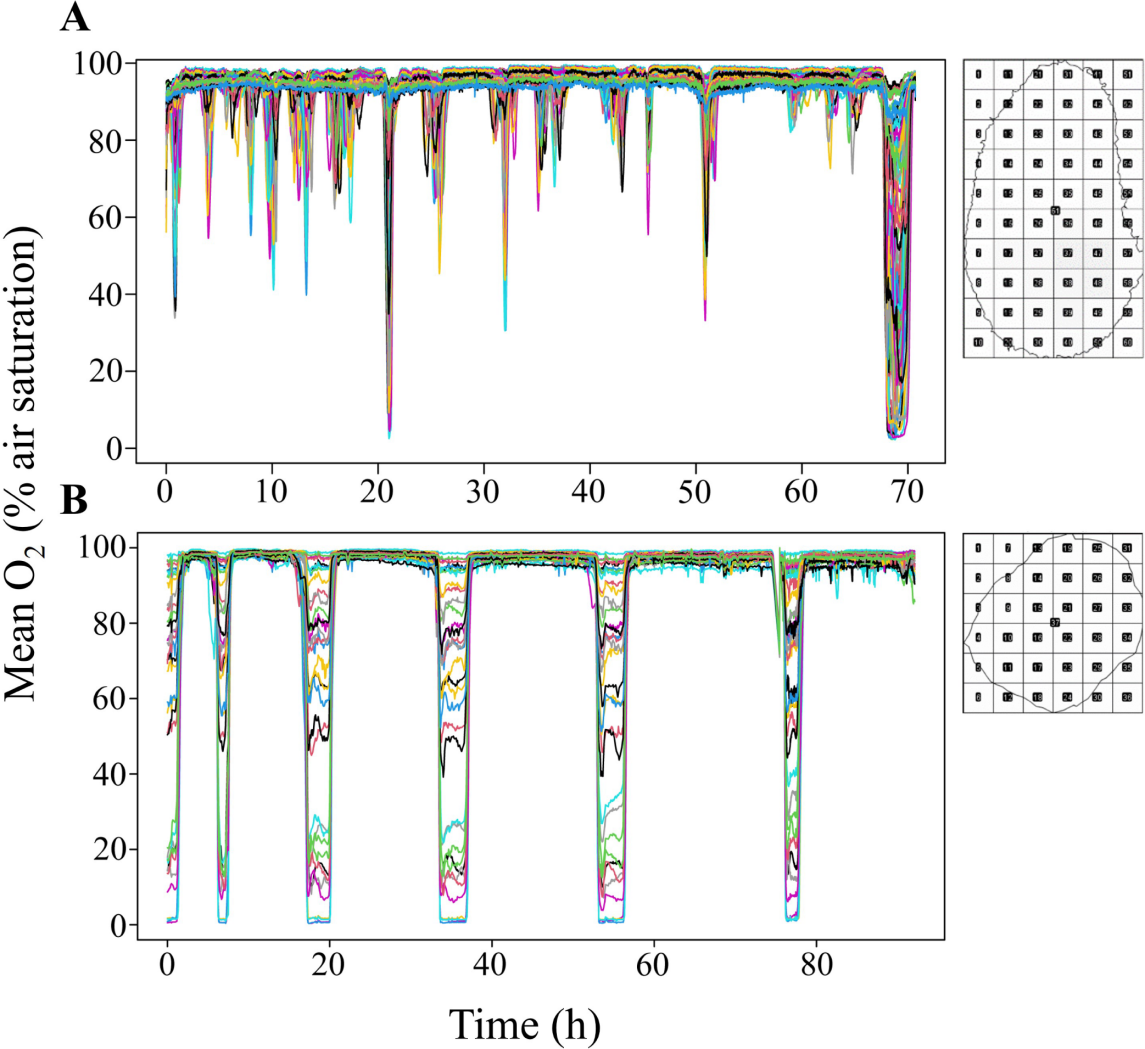
Time-series measurements of mean oxygen levels in individual segments of the sponge base for **(A)** *H. rosea* (*ID*2) and **(B)** *H. panicea* (*ID*3) explants. Each colored line represents the oxygen dynamics in a distinct spatial segment of the sponge base over the observation period. Corresponding segment maps on the right show the spatial arrangement of the analyzed regions. The black line indicates the contour of the sponge base grown on the oxygen-sensitive planar optode.

Time-series measurements of mean oxygen levels in individual segments of the sponge base revealed that deoxygenation is differently synchronized in the examined *H. rosea* and *H. panicea* explants (**Figure 4**). In *H. rosea*, deoxygenation occurred mostly asynchronously, with oxygen levels dropping in individual segments at irregular intervals and with varying magnitudes (**Figure 4A**). No coordinated patterns of oxygen fluctuations were observed between segments, indicating localized, i.e. segment-specific oxygen dynamics.

In contrast, *H. panicea* exhibited highly synchronous oxygen reductions across all segments (**Figure 4B**). During these deoxygenation events, oxygen levels in most segments dropped sharply, often approaching anoxia (< 1% air saturation), followed by a coordinated recovery to near ambient oxygen levels. The consistent timing and synchrony of these fluctuations suggests a systemic mechanism coordinating intrinsic (and periodic) deoxygenation in *H. panicea*.

### 3.3 Characteristic spatiotemporal patterns in *H. rosea*

**Figure 5:**
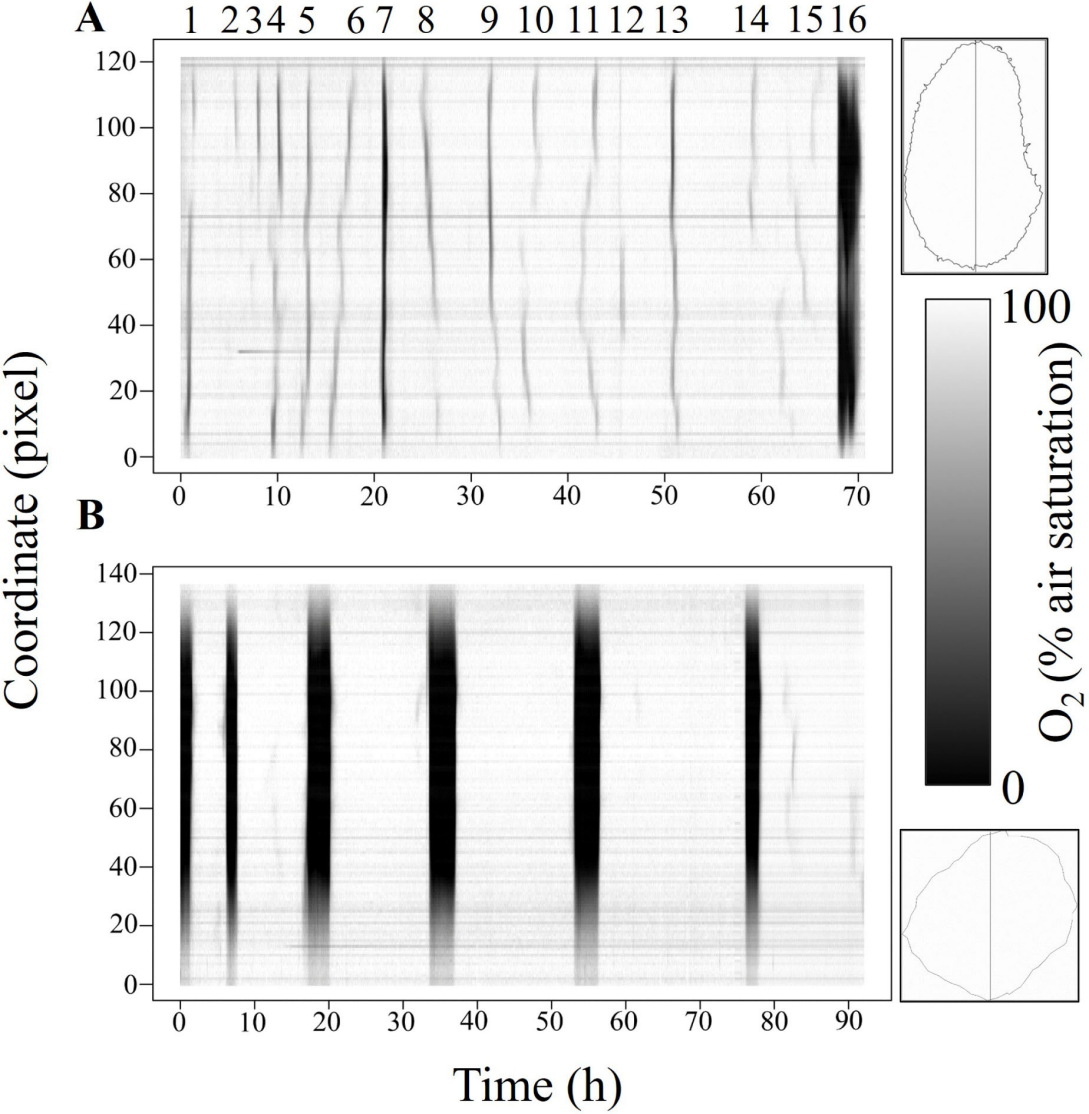
Spatiotemporal patterns of internal deoxygenation in single-osculum demosponge explants. Space-time plots show oxygen saturation measured in pixels along a vertical transect bisecting the base of representative **(A)** *H. rosea* (*ID*2) and **(B)** *H. panicea* (*ID*3) individuals. The x-axis denotes time, while the y-axis represents the spatial position along the transect (resolution: ~100 μm/pixel). Internal oxygen saturation is encoded as a grayscale gradient ranging from fully oxygenated (white) to anoxic (black). Numbered annotations in **(A)** mark deoxygenation events (#1–#16) observed in *H. rosea* (*ID*2), including representative examples such as recurring propagating waves (e.g. events #8 and #9), spiral-like patterns (e.g. event #11, Video S1) and unidirectionally advancing fronts of deoxygenation (see Section 3.3 for detailed descriptions). Synchronous deoxygenation events, as predominantly observed in *H. panicea* (**B**), appear as broad bands with central anoxia and increasing oxygen saturation towards the explant periphery. The exact transect positions relative to the explant contours are depicted to the right of each respective panel. Corresponding plots for all other individuals are provided in **Supplementary Figure 3**.

Space-time plots reveal distinct spatiotemporal patterns in oxygen dynamics for *H. rosea* and *H. panicea* (**Figure 5**). Representative examples for both species are depicted in **Figure 5**. In *H. rosea*, we identified several recurring patterns, ranging from irregular, weakly ordered dynamics (e.g. event #12, **Figure 5**) to more structured, persisting cycles of local oxygen depletion and recovery (e.g. events #8 and #9, **Figure 5**). Transitions between these patterns were common, and in some instances, events evolved seamlessly from one form into another (e.g. events #2 - #4 and #15 - #16 in **Figure 5**), Such transitions marked changes in the spatial extent and temporal regularity of oxygen dynamics over time (**Figure 5**; **Supplementary Table 1**).

The “*synchronous pattern*”, characteristic for *H. panicea*, and also observed in some cases in *H. rosea* (event #16, **Figure 5A**), is marked by coordinated deoxygenation across the entire sponge base (**Figures 5A, B**). This pattern leads to acute, uniform deoxygenation of the sponge interior, forming concentric bands with oxygen isopleths and anoxia concentrated in the center.

In *H. rosea*, we recorded additional versatile and complex patterns, some of which are illustrated in the supplementary videos. One prominent pattern involved recurring, propagating waves of oxygen reduction that moved across the sponge base (e.g. events #8 - #11; **Figure 5**). These waves often formed nearly identical strips in space-time plots (see events #8 & #9 and #10 & #11), where diagonally oriented bands indicate the directional propagation of low-oxygen regions. Occasionally, the waves formed spiral-like patterns of low oxygen (i.e. “*regular rotating spiral waves*”, see e.g. event #11, **Supplementary Video S1**), propagating in both clockwise and counterclockwise directions. These spirals, one or multiple in number, frequently fused, split, and migrated across the sponge base. Spirals also eventually disappeared and/or spontaneously transitioned into the synchronous pattern (events #15 - #16, **Supplementary Video S2**). In other cases, such as event #5, we observed a distinct advancing boundary of oxygen depletion resembling an “*unidirectional wave*” (**Supplementary Video S3**), appearing as single, continuous front in the space-time plot. These findings underscore the diversity in spatiotemporal oxygen dynamics in demosponges, highlighting fundamental differences in oxygen regulation dynamics in sponges.

## 4 Discussion

### 4.1 Distinct oxygen dynamics in single-osculum explants of two demosponge species

Our results demonstrate that both *Halichondria panicea* and *Haliclona rosea* exhibit dynamic internal oxygenation patterns that vary in both space and time. These deoxygenation/oxygenation dynamics point to a level of physiological regulation that is more nuanced than previously appreciated in sponges. Indeed, internal tissue oxygen levels are actively modulated in ways that create spatially structured and temporally variable oxygen microenvironments. Modulation and subsequent oxygen dynamics differ markedly between the two species. Thus, *H. rosea* exhibited localized and asynchronous deoxygenation events, indicating a high degree of area-specific regulation. In contrast, *H. panicea* showed synchronized, large-scale cycles of oxygen depletion and recovery, consistent with coordinated, regulatory mechanisms. Despite these differences, both species spent a similar proportion (~26%) of the observation period in oxygen-reduced states, suggesting a shared capacity, and likely necessity, for internal oxygen regulation.

The contrasting oxygen dynamics observed here are likely linked to different contractile behaviors of the two sponge species. Contractions of the aquiferous system, including the inhalant pores (i.e. ostia) and exhalant osculum, suppress waterflow generated via pumping, leading to oxygen loss due to respiration in the sponge interior (Hoffmann et al., 2008; Kumala and Canfield, 2018). In *H. panicea*, contractions appear to be synchronized across the entire body (Goldstein et al., 2020) and are coupled with osculum closure. In the subsequent absence of internal water flow, oxygen levels decline throughout the sponge, leading to pronounced deoxygenation at the explant base and the development of central anoxia, consistent with previously predicted whole-body oxygen depletion in this species (Kumala and Canfield, 2018; Kumala et al., 2021).

Other contraction patterns may explain the more finely controlled oxygen dynamics in *H. rosea*. Demosponges exhibit a wide repertoire of behaviors observed along the surface of the sponge. These include spasms, ripples, and peristaltic waves of contractions (Elliott and Leys, 2007; Harrison et al., 2024) that can constrict water canals and temporarily reduce ventilation in distinct body regions (cf. Schläppy et al., 2010b; Kumala et al., 2021). Localized, but mobile, reductions in ventilation may explain some of the spatially limited but mobile deoxygenation patterns in *H. rosea*. The restriction in water flow may lack the areal extent, the time, and the degree of restriction to allow complete oxygen drawdown characteristic of whole-body deoxygenation. Consistent with this, the lowest oxygen levels in *H. rosea* tend to occur primarily during prolonged deoxygenation events.

Structural differences between the two species may underlie the contrasting behavior and associated oxygen patterns. In demosponges, pinacocytes mediate contractile behaviors (Nickel et al., 2011; Leys, 2015; Ruperti et al., 2024), and their epithelial arrangement affects whether water flow through the aquiferous system is locally or globally modulated. In *H. rosea*, choanocyte chambers are spatially separated from the (poorly developed) mesohyl by a pinacocyte epithelium extending from the incurrent canals (Langenbruch and Jones, 1990). The contractile activity of pinacocytes along discrete choanocyte chambers or canals may permit modulation of water flow at the level of individual choanocyte chambers and canal segments, thereby locally altering ventilation and oxygen availability. Additionally, the spatial separation of choanocyte chambers from the mesohyl in *H. rosea* may impede the transmission of systemic signals (e.g. neurotransmitter-like molecules or calcium waves) across the sponge body, limiting coordinated behavior (Leys and Meech, 2006; Elliott and Leys, 2010).

In contrast, the integration of choanocyte chambers into the mesohyl in *H. panicea* may facilitate the transmission of chemical or ionic signals, enabling whole body-coordinated contractions (Goldstein et al., 2020; Kumala et al., 2021). Ciliated (mechano-) sensory cells lining the sponge osculum are likely involved in coordinating such global behavior (Ludeman et al., 2014). Therefore, osculum closure may be central to the whole-body deoxygenation cycles (Kumala et al., 2021), whereas localized deoxygenation events may arise from osculum-independent, locally integrated mechanosensory and contractile activity within discrete canals or choanocyte chambers (cf. Musser et al., 2021). Such localized contractions lead to spatially heterogeneous reductions in ventilation and oxygen availability, generating temporally dynamic oxygen microenvironments in sponges with open oscula, as previously suggested for other sponge species such as *Theonella swinhoei* (Lavy et al., 2016).

Notably, the capacity for both local and global contractions likely exists in both species, but the dominant behaviors expressed under specific environmental conditions differ. In *H. panicea*, peristaltic contraction waves can occur during internal cleaning, while whole-body contractions can be triggered by particle overload and other environmental cues, but also occur spontaneously as part of intrinsic behavioral rhythms (reviewed in Goldstein and Funch, 2022; Goldstein et al., 2024). During our experiments, where explants were likely maintained under relatively low particle load (Kumala and Riisgård, 2024), only whole-body contractions were observed in *H. panicea* (Kumala et al., 2021). This suggests that *H. panicea* may prioritize global regulation strategies under the provided experimental conditions. Conversely, *H. rosea* primarily exhibited asynchronous events but occasionally displayed coordinated, body-wide oxygen depletion, indicating that it too can perform synchronized contractions. Together, these observations suggest that both sponge species likely share a molecular and behavioral toolkit enabling a range of contractile behaviors but express different behaviors under similar experimental conditions. Given the limited number of *H. rosea* individuals examined, additional replication is needed to evaluate the consistency of the behavior and associated oxygen dynamics in this species. Nevertheless, the overall contrast between localized and asynchronous deoxygenation dynamics in *H. rosea* and coordinated whole-body oxygen depletion in *H. panicea* was qualitatively consistent across the examined individuals in the present study. Whether rhythmic deoxygenation arises from intrinsic (e.g. Reiswig, 1971) or external triggers remains unresolved, but the contrasting oxygen patterns observed in *H. rosea* and *H. panicea* suggest differences in their regulatory behavior. *H. rosea* achieves fine-scale, region-specific control of tissue deoxygenation, whereas the coordinated, whole-body contractions of *H. panicea* function as a global regulatory mechanism that prominently involves osculum-mediated suppression of ventilation. Further studies with larger sample sizes are needed to determine whether these patterns represent consistent species-specific regulatory strategies or context-dependent behavioral expression.

### 4.2 Oscillations in oxygen dynamics

Oscillations describe recurring variations of biological and chemical variables around a central value or between distinct states and are pervasive features of most organisms. Oscillations govern critical physiological processes ranging from intracellular signaling and cell motility to metabolism, growth and cell division (Goldbeter, 2008; Xiong and Garfinkel, 2023). Oscillatory behavior enables physiological systems to prevent desensitization, limit the accumulation of potentially harmful metabolites, and coordinate processes that would otherwise be incompatible or difficult to synchronize (Xiong and Garfinkel, 2023). Oxygen may play a central role in such dynamics, as it directly links aerobic metabolism, cellular/tissue redox homeostasis, and signaling pathways.

The oxygen dynamics observed in the present study suggest oscillatory behavior in oxygen regulation in both demosponge species. In both sponges, oxygen patterns are characterized by repeated cycles of oxygen reduction and recovery, yet they differ markedly in their spatial and temporal organization. These distinct oscillatory patterns are likely related to behavioral control mechanisms that rely on the coordination of mechanical as well as chemical signals in space and time (Leys et al., 2024). In demosponges, signaling pathways involving calcium ions (Ca^2+^) and ATP are directly implicated in contraction-expansion dynamics (Colgren and Nichols, 2022; Ho et al., 2025). Notably, these molecules act as key oscillating components in many other biological systems, where they can function individually or as coupled oscillatory networks (e.g. Ridgway and Durham, 1976; Evans and Sanderson, 1999).

Oscillatory Ca^2+^ and ATP dynamics are known to modulate rhythmic behaviors in diverse eukaryotic systems. For instance, in the slime mold *Physarum polycephalum*, spatiotemporal oscillations in Ca^2+^ concentration and ATP availability control actomyosin relaxation-contraction cycles that drive pulsatile migration of the organism (Ridgway and Durham, 1976; Boussard et al., 2021 and references therein). Whether intracellular Ca^2+^ oscillations are phase-locked to sponge contraction cycles remains to be demonstrated. However, Ca^2+^ signaling acts upstream of myosin light-chain kinase (MLCK), which is known as central regulator of actomyosin contractility. Consequently, fluctuations in intracellular Ca^2+^ levels are expected to be tightly coupled in time to actomyosin-mediated tissue contractions (Colgren and Nichols, 2022; but see Ruperti et al., 2024). Given the deep evolutionary conservation of contractile epithelial cell types and associated mechanosensitive signaling pathways (Musser et al., 2021; Colgren and Nichols, 2022; Ruperti et al., 2024; Brunet, 2023), it is plausible that oscillatory Ca^2+^ and potentially other signaling molecule dynamics contribute to the contraction and associated deoxygenation-reoxygenation cycles observed in sponges. Oscillations in intracellular Ca^2+^ and related signaling pathways occur on physiologically relevant timescales ranging from seconds to minutes and, in some systems, hours, which overlap with rhythmic (contractile) behaviors observed in many organisms, including sponges (Uhlén and Fritz, 2010 and references therein; Boussard et al., 2021; Ho et al., 2025). In some systems, including mammals, such oscillatory signaling is also linked to changes in oxygen utilization and availability (Hu and Ziegelstein, 2000; Du et al., 2014), further supporting a functional coupling between rhythmic activity and oxygen dynamics.

A major advantage of adopting oscillatory behavior in sponges is that it maintains a dynamic, non-steady state allowing rapid transitions between functional modes in response to sudden environmental changes. This mode of regulation may be particularly important in organisms that lack a nervous system and true muscles, such as sponges, where oscillatory signaling may be essential for coordinating activity across cells and tissues. Oscillatory, diffusible, signal molecules, including Ca^2+^, cAMP, and reactive oxygen species (ROS), typically self-organize into spatial patterns that coordinate tissue-scale behavior (Winfree, 1972; Novák and Tyson, 2008).

Spatiotemporally complex patterns, such as those observed in *H. rosea*, are also found in other biological systems. For instance, in cellular slime molds, spiral waves of cAMP govern cell aggregation in *Dictyostelium discoideum* (Tyson et al., 1989), while oscillations of Ca^2+^ and ATP coordinate cytoplasmic streaming and rhythmic migration in *P. polycephalum* over relatively large spatial scales (Yoshiyama et al., 2010). In contrast, the global oxygen patterns observed in *H. panicea* suggest spatially synchronized signaling, in which oscillatory signals are integrated across the entire organism, analogous to metabolic oscillations or circadian cycles (Goldbeter, 1997; Tu and McKnight, 2006). While such a pattern could, in principle, arise from rhythmic osculum closure alone, osculum dynamics are tightly coupled to coordinated contractions throughout the aquiferous system, including ostia as well as in- and excurrent canals (Goldstein et al., 2020). Mechanical coupling among sponge pinacocytes likely acts as the primary driver of synchronized cellular signaling, producing coordinated contractions that manifest as whole-body oscillations (Ruperti et al., 2024).

Whether cytoplasmic streaming, cell migration, or tissue remodeling contribute directly to or overlap with the oxygen dynamics observed in sponges remains to be determined. However, such processes are known to play important roles in movement (Bond, 1992), regeneration (Ereskovsky et al., 2020, Ereskovsky et al., 2021), and cleansing mechanisms in sponges (Funch et al., 2023) and may hence interact with oxygen regulation observed in our sponges. In fact, oxygen and its reactive oxygen species can act as signaling molecules in other animals, where they regulate processes such as cell differentiation, wound healing and tissue regeneration (Rodriguez et al., 2008; Bartz and Piantadosi, 2010; Hammarlund et al., 2018). Thus, oxygen dynamics in sponges may represent active components of broader oscillatory networks synchronizing fundamental developmental and regenerative processes that cannot occur simultaneously in sponges due to conflicting physiological requirements, such as active water-pumping versus tissue remodeling under reduced oxygen conditions.

In many organisms, internal oxygen levels oscillate in accordance with physiological cycles such as feeding, activity, or rest (Tu and McKnight, 2006; Adamovich et al., 2019). For instance, circadian rhythms in mammals such as mice and rats impose daily oscillations in tissue oxygenation that are controlled by the molecular clock and modulated by behavioral cycles of feeding and activity (Adamovich et al., 2019; Emans et al., 2017). Oxygen fluctuations, including transient and spatially restricted hypoxia, are increasingly recognized as key components of endogenous metabolic and circadian regulation (e.g. Adamovich et al., 2017). Therefore, the oxygen dynamics observed here, particularly in *H. panicea*, may represent an additional manifestation of the circadian-like regulation previously shown in sponges at both molecular (Jindrich et al., 2017) and organismal levels (Flensburg et al., 2022).

In some demosponges, contraction behavior and respiration exhibit diel modulation linked to environmental light cycles. For example, the demosponge *Tethya wilhelma* displays pronounced diel contraction-expansion behavior, including sunrise-induced whole-body contractions that nearly diminish under constant darkness (Flensburg et al., 2022). In some coral reef sponges, such as *T. swinhoei*, diel fluctuations in respiration, including elevated nocturnal metabolic activity, have been reported (Moskovich et al., 2023). Because our experiments were conducted in the absence of a natural light-dark cycle, the influence of photoperiod on contraction behavior and internal oxygen dynamics could not be assessed in the present study. However, rhythmic contractions and recurring deoxygenation events were still observed, particularly in *H. panicea*. Moreover, the duration and periodicity of the observed behavior were comparable to previous observations in *H. panicea* explants maintained under constant light conditions (Kumala et al., 2017; Kumala and Canfield, 2018), as well as to *in situ* contraction dynamics reported for other demosponge species under natural environmental conditions, including light-dark cycles (e.g. Reiswig, 1971; Harrison et al., 2024). This suggests that the observed oxygen dynamics reflect intrinsic physiological behavior, yet future studies incorporating controlled light-dark cycles are needed to determine how the natural photoperiod influences the frequency, duration and magnitude of these deoxygenation events.

Oxygen oscillations may prevent chronically elevated intracellular oxygen concentrations, which would otherwise promote excessive ROS production and oxidative stress due to finite enzymatic antioxidant capacity (Murphy, 2009; Halliwell and Gutteridge, 2015). Low levels of ROS are continuously generated as by-products of aerobic metabolism, but intracellular ROS can accumulate during periods of elevated metabolic activity or physiological stress, such as dehydration (França et al., 2007). Temporal reductions in oxygen availability, including internal hypoxia or anoxia, can constrain aerobic metabolism, thereby aligning metabolic demand with oxygen supply, limiting excess ROS formation, and potentially facilitating tissue repair processes. This mechanism may be particularly relevant for intertidal sponge species inhabiting photic-zone environments, such as *H. panicea* and *H. rosea*, which are episodically exposed to oxygen-supersaturated seawater as well as air during low tide. Moreover, sponges are known to actively produce and release substantial amounts of ROS (e.g. superoxide) into the surrounding water (Taenzer et al., 2023) via processes linked to immune-related functions such as phagocytosis (Gordeeva et al., 2006) and enzymes homologous to NADPH oxidases in other animals (Hewitt and Degnan, 2022). However, whether the oscillatory oxygen dynamics directly reduce the risk of sustained oxidative stress or contribute to redox homeostasis in sponges remains a hypothesis that requires experimental testing. In this context, the observed oxygen dynamics may contribute to balancing metabolic activity and ROS production while still permitting ROS-mediated signaling and defense functions.

Intra- and interspecies-specific differences in metabolic rates may therefore play a central role in shaping the frequency, amplitude and spatial extent of the observed oxygen oscillations. Metabolic (i.e. aerobic respiration, *R*) rates in sponges vary within a factor of about 30 among species (i.e. from 0.23 to 6.7 µmol O_2_ h^-1^ mL^-1sponge^; reviewed in Osinga et al., 1999). During periods of pumping cessation, high respiration rates are expected to promote the expansion of oxygen-depleted regions, which could contribute to the global deoxygenation observed in *H. panicea* (Kumala and Canfield, 2018; Kumala et al., 2021). Conversely, localized constriction of the water canal system, together with lower respiration, may confine oxygen depletion to discrete tissue domains, potentially giving rise to the complex spatiotemporal patterns observed in *H. rosea*.

Both ambient and internal oxygen availability can directly modulate sponge aerobic metabolism once critical threshold levels are reached (Micaroni et al., 2021; Kumala et al., 2023; Riisgård, 2024). In *H. panicea*, for example, respiration remains nearly constant over a wide range of external oxygen levels but decreases markedly below ~20% air saturation. Thus, we hypothesize that oxygen-dependent changes in respiration alter both the extent and persistence of oxygen-depleted regions in the sponge interior, thereby reshaping internal oxygen dynamics under varying environmental oxygen conditions. Reduced ambient oxygen levels may induce behavioral shifts that transition globally synchronized deoxygenation towards more spatiotemporally heterogeneous oxygen patterns in *H. panicea*.

### 4.3 Evolutionary implications of oxygen regulation in early metazoans

Today, oxygen constitutes ~21% of Earth’s atmosphere and is readily available in most environments. However, Earth’s atmospheric oxygen levels have varied dramatically through geological time, including prolonged intervals of low and variable oxygen levels (Lenton, 2003; Canfield et al., 2021). Animal life diversified under these variable oxygen conditions, and early-branching metazoans evolved during periods when oxygen levels were significantly lower than today (Lenton et al., 2014; Mills et al., 2014). Sponges probably represent one of the earliest extant animal lineages (e.g. Schultz et al., 2023), with fossil and molecular evidence dating their origins 750–800 Ma ago (e.g. Dohrmann and Wörheide, 2017), when atmospheric oxygen may have ranged somewhere from 1 to 20% of modern levels (Canfield et al., 2021; Krause et al., 2022).

Regulating oxygen temporally and spatially was likely less critical in early low-oxygen environments, where ambient oxygen concentrations would have kept intracellular oxygen levels low by default. Under such conditions, mitochondrial respiration could proceed at low oxygen tensions (Mentel et al., 2014; Mills et al., 2014), and reactive oxygen species (ROS) production from aerobic metabolism would have been limited (Turrens, 2003 and references therein). The need for active oxygen regulation may therefore have been reduced, and regulatory strategies associated with internal oxygen cycling may have been diminished, absent, or fundamentally different from those operating under modern oxygen conditions. Cellular (e.g. enzymatic) mechanisms for managing oxygen likely evolved alongside aerobic metabolism, whereas more elaborate tissue-level oxygen regulation emerged later with multicellularity and increasing body size (Bernroitner et al., 2009; Hammarlund et al., 2018, Hammarlund et al., 2020b; Hammarlund, 2020a).

Rising and variable oxygen availability during later Earth history likely imposed new constraints on early animals. Higher intracellular oxygen concentrations promote ROS formation during mitochondrial respiration (reviewed in Turrens, 2003), creating a trade-off between efficient aerobic metabolism and limiting oxidative damage (e.g. Bekker et al., 2004). Early metazoans were therefore required to reconcile fundamentally incompatible redox conditions within the same organism (e.g. Mentel et al., 2014), a challenge that may have favored cycling between oxic and hypoxic/anoxic states. Such internal redox heterogeneity would also have facilitated early metabolic interactions and symbioses, allowing microorganisms with distinct oxygen requirements to coexist within host tissues. Modern sponges host diverse microbial communities spanning aerobic, microaerophilic, and anaerobic metabolisms, and transient oxygen gradients or oscillatory redox conditions are thought to promote this metabolic partitioning (e.g. Zhang et al., 2019). Spatiotemporal shifts in internal oxygenation, modulated by global and localized contractile behavior, may shape the activity and spatial distribution of metabolically distinct microorganisms within the sponge holobiont (Kumala et al., 2021 and references therein). In this context, oxygen regulation may represent an ancestral feature of modern sponges, assuming that the aquiferous system (Suarez and Leys, 2022), epithelial tissues (Leys, 2007; Leys et al., 2009), and mechanisms for coordinated control of water flow (Leys, 2015; Leys et al., 2019) emerged early in sponge evolution.

Large sponge body plans likely evolved late in sponge evolution, not because of low environmental oxygen limiting sponge size, but with the emergence of sponge modularity, i.e. the ability to assemble multiple aquiferous units into a large sponge body (Kumala et al., 2023). The earliest known sponges from the early Cambrian were likely solitary, single-osculum forms (Wood et al., 1992), and fossil evidence suggests that Cambrian sponges possessed the ability to contract their osculum in response to external stimuli (Luo et al., 2025). Given our results, this implies that behavioral control of internal oxygenation via osculum closure may be an ancestral feature of early Cambrian sponges. In modern, multi-modular sponges, asynchronous osculum closure enables individual aquiferous units to become transiently isolated from other aquiferous units (Riisgård et al., 2016), leading to module-specific differences in internal oxygenation (Kumala et al., 2021). Importantly, our observations in single-osculum *H. rosea* show that fine-scale oxygen regulation can occur within a single aquiferous module, generating spatiotemporally dynamic oxygen patterns even in the absence of multi-modular body organization and independent of osculum closure. This indicates that internal oxygen heterogeneity does not require osculum-defined modularity but can arise from coordinated contractile behavior across epithelial cell populations (Ruperti et al., 2024; Brunet, 2023), creating spatially distinct oxygen microenvironments within the individual module. Together, these findings suggest that behavioral oxygen control likely predates the evolution of modular sponge body plans and perhaps also the emergence of larger aquiferous modules and body sizes (Kumala et al., 2023).

Given that early metazoans evolved under markedly lower atmospheric O_2_ concentrations, the development of fine-scale mechanisms for perceiving and responding to ambient oxygen levels was likely pivotal for animal tissues to adapt to the oxic realm (Hammarlund et al., 2018, Hammarlund et al., 2020b). Oxygen-sensitive pathways have been crucial in shaping multicellular life: low oxygen governs stem cell fate, tissue regeneration (Hammarlund et al., 2018, Hammarlund et al., 2020b) and the development of nervous (Panchision, 2009) or cardiovascular systems (Fisher and Burggren, 2007; Cordeiro and Tanaka, 2020). Central to these processes is the Hypoxia-Inducible Factor (HIF) pathway, a primary molecular oxygen-sensing mechanism in animals, which responds to changes in intracellular O_2_ through O_2_-dependent enzymatic (i.e. hydroxylase) reactions and downstream transcriptional control (Semenza, 2007). HIF-controlled pathways are deeply conserved across most animals (Hammarlund et al., 2018), and maintain physiological oxygen homeostasis despite environmentally or metabolically driven oxygen fluctuations (Loenarz et al., 2011). Efficient oxygen sensing mechanisms, particularly those regulated by HIF-α, are predicted to have conferred a selective advantage during animal diversification in the Neoproterozoic-Paleozoic by mitigating severe physiological stress associated with diel benthic oxygen fluctuations (Hammarlund et al., 2025). Given our finding that sponges actively shape their internal oxygenation, one might therefore expect similar (i.e. HIF-related) oxygen-sensitive molecular pathways to operate in sponges.

However, current genomic and transcriptomic evidence for canonical HIF-mediated oxygen sensing in sponges remains equivocal. Sponges, together with ctenophores, appear to lack key features of the HIF pathway, including bilaterian hydroxylation motifs required for HIF regulation (Mills et al., 2018; Hammarlund et al., 2018), whereas putative homologs of HIF-pathway genes (i.e. HIF, PHD, VHL and FIH) have more recently been identified in the demosponge *Amphimedon queenslandica* (Song et al., 2022). This indicates that sponges, and likely ctenophores, may sense and respond to low oxygen differently than most other animal phyla (Mills et al., 2014, Mills et al., 2018; Hammarlund et al., 2025).

Some demosponges, such as *T. wilhelma*, show little or no significant transcriptional changes even under very low (0.25% air saturation) ambient oxygen conditions. Nevertheless, *T. wilhelma* alters its contraction behavior in response to low oxygen availability (Mills et al., 2018). These sponges switch from regular full body contractions to more localized contractions at oxygen levels ≤1.86% AS. Analogous to the dynamics observed in our study, this behavioral shift reflects a transition from global to more spatiotemporarilly heterogeneous oxygenation states in *T. wilhelma*. Together, these observations suggest that sponge responses to ambient or internal oxygen depletion may rely on alternative, perhaps more ancestral mechanisms of oxygen sensing and transcriptional regulation (Mills et al., 2018; Hammarlund et al., 2018; Song et al., 2022). Potential alternative mechanisms for oxygen sensing and regulation may for instance occur at the mitochondrial level, where sulfide accumulation under anoxia could signal oxygen depletion, while subsequent detoxification proceeds through conserved cellular pathways (Mills et al., 2018 and references therein). In addition, recent work suggests that, in the absence of HIF, internal oxygen oscillations in sponges may represent a unique adaptation for regulating cell fate and tissue maintenance, as anoxia typically promotes stemness while oxic conditions facilitate differentiation (Hammarlund et al., 2018, Hammarlund et al., 2020b, Hammarlund et al., 2025). Such behavioral control of internal oxygenation may have contributed to sponge diversification despite the apparent absence of a HIF-α pathway, which in Bilateria regulates cell differentiation and plays a key role in tissue homeostasis (Hammarlund et al., 2025). In this way, rhythmic modulation of internal oxygenation may have provided sponges with an alternative means to cope with fluctuating redox conditions in the environment, potentially reducing reliance on HIF-α-mediated oxygen sensing proposed to facilitate diversification in other animal lineages (Hammarlund et al., 2025).

In sponges, oxygen-dependent regulatory processes seem particularly relevant when internal oxygen supply is disrupted, such as during tissue regeneration and reconstruction of the aquiferous system following injury or (sexual and asexual) reproduction (Ereskovsky et al., 2020, Ereskovsky et al., 2021). A compelling example comes from regenerating sponge explants, which typically experience extensive internal anoxia before their aquiferous system is re-established (Hoffmann et al., 2005a). In *H. panicea* explants, several genes belonging to the main molecular machinery of blood vessel formation (or vasculogenesis) known for vertebrates were upregulated during regeneration, including vascular cell adhesion molecule 1, E-selectin and the key vasculogenic/angiogenic regulator VEGF-R1 (Riesgo et al., 2022). Transcription of these genes is classically regulated by hypoxia in vertebrate tissues (e.g. Zünd et al., 1996; Risau, 1997), and their upregulation in oxygen-depleted explants lacking a functional aquiferous system indicates that sponge tissues may similarly deploy these gene repertoire to rebuild their water canal system when oxygen supply via internal water-pumping is compromised. In this sense, reconstruction of the aquiferous system in sponges may represent an analogue of vasculogenesis, in which reduced internal oxygen acts as signal that initiates regenerative processes aimed at restoring oxygen supply to tissues. Together, these observations indicate that behaviorally generated oxygen fluctuations, including transient hypoxia or anoxia, might represent an integral component of how sponge tissues and the aquiferous system are remodeled and maintained. Such internal oxygen dynamics likely have far-reaching implications for sponge development, embryogenesis and metabolic organization, and may represent an ancient mode of regulation that evolved early in animal evolution. Investigating whether spatiotemporal oxygen regulation occurs in other early-diverging animal groups, such as Ctenophora, Placozoa, or Cnidaria, remains a key priority for future research.

## 5 Conclusion

The rise in atmospheric oxygen enabled the evolution of larger animal body sizes and necessitated the development of mechanisms to manage oxygen toxicity and ensure adequate oxygen distribution. Most animals evolved compartmentalized body plans, specialized tissues, organs, and circulatory systems that dynamically regulate oxygen delivery in response to oxygen demands. Here, we show that sponges, known as one of the simplest metazoans, can modulate internal oxygen levels in both space and time. Distinct deoxygenation-reoxygenation patterns in sponges are generated by intrinsic oscillatory contractile behaviors that are likely coordinated by the interplay between intra- and intercellular signaling molecules, such as ATP, Ca^2+^, or ROS, and actomyosin. Global and local modulation of internal water flow likely provide for fine- and large-scale patterns in oxygen distribution. These findings suggest that rhythmic modulation of internal oxygen levels, whether spatially complex or spatially uniform, can arise in the absence of nerves, muscles, or true circulatory systems.

Taken together, our results suggest that the fundamental principles of flow regulation arose early in animal evolution. Oxygen regulation in sponges reflects deep evolutionary heritage. The ability to generate internal oxygen gradients, respond to them, and actively modulate tissue oxygenation may have been pivotal to early animal evolution as environmental oxygen became increasingly available and variable. These ancient regulatory capacities likely informed the later evolution of centralized vascular systems, neuronal control of tissue perfusion, and the sophisticated oxygen homeostasis mechanisms that characterize modern animals.

## Data Availability

The raw data supporting the conclusions of this article will be made available by the authors, without undue reservation.
