# Supplementary material for: Oxygen regulation in the earliest animals: spatiotemporal resolution of oxygen dynamics in single-osculum Halichondria panicea and Haliclona rosea sponges
Source: Front Physiol. 2026 Jul 29;17:1849630. doi: 10.3389/fphys.2026.1849630 (PMC13461416; doi:10.3389/fphys.2026.1849630)
Supplement: Supplementary file 1 [file DataSheet1.docx]

*The following supplement accompanies the article*

**Oxygen regulation in the earliest animals: spatiotemporal resolution of oxygen dynamics in single-osculum *Halichondria panicea* and *Haliclona rosea* sponges**

Lars Kumala^1,2,^***, Malte Thomsen^1,2^, and Donald E. Canfield^1,3^

*^1^Nordcee, Department of Biology, University of Southern Denmark, 5230 Odense M, Denmark*

*^2^Marine Biological Research Centre, University of Southern Denmark,*

*DK-5300 Kerteminde, Denmark*

*^3^Danish Institute of Advanced Study (DIAS), University of Southern Denmark, 5230 Odense M, Denmark*

**Correspondence: Lars Kumala, E-mail: kumala@biology.sdu.dk*

**Table S1.** Overview of events with substantial changes in O_2_ (#1 to #28, Figures 1 & S1) measured at the base of single-osculum *Haliclona rosea* (*ID*1 to *ID*2) and *Halichondria panicea* sponges (*ID*1 to *ID*4) grown on oxygen-sensitive planar optodes. The table includes event duration (*D*), time interval (∆*t*) until the subsequent event, range of lowest oxygen levels ([O_2_]) reached during the event, time (*t*) at the lowest oxygen levels, and the proportion of the total event duration spent at the lowest oxygen levels (*t_D_*).

| *Species* | *ID* | *Event no.*  # | *D*  (min) | ∆*t*  (min) | [O2]  (% AS) | *t*  (min) | *t_D_*  (%) |
| --- | --- | --- | --- | --- | --- | --- | --- |
| *H. rosea* | 1 | 1 | 50 | - | <50 & >40 | 8 | 16.0 |
|  |  | 2 | 105 | 73 | <50 & >40 | 46 | 43.8 |
|  |  | 3 | 30 | 69 | <60 & >50 | 20 | 66.7 |
|  |  | 4 | 86 | 16 | <20 & >15 | 3 | 3.5 |
|  |  | 5 | 142 | 99 | <15 & >10 | 8 | 5.6 |
|  |  | 6 | 84 | 38 | <25 & >20 | 2 | 2.4 |
|  |  | 7 | 95 | 58 | <50 & >40 | 3 | 3.2 |
|  |  | 8 | 19 | 47 | <60 & >50 | 11 | 57.9 |
|  |  | 9 | 10 | 80 | <60 & >50 | 1 | 10.0 |
|  |  | 10 | 91 | 88 | <25 & >20 | 6 | 6.6 |
|  |  | 11 | 37 | 353 | <40 & >30 | 9 | 24.3 |
|  |  | 12 | 64 | 219 | <25 & >20 | 6 | 9.4 |
|  |  | 13 | 9 | 24 | <70 & >60 | 9 | 100.0 |
|  |  | 14 | 71 | 471 | <40 & >30 | 4 | 5.6 |
|  |  | 15 | 7 | 418 | <60 & >50 | 2 | 28.6 |
|  |  | 16 | 85 | 34 | <5 & >1 | 11 | 12.9 |
|  |  | 17 | 61 | 382 | <20 & >15 | 2 | 3.3 |
|  |  | 18 | 39 | 4 | <50 & >40 | 10 | 25.6 |
|  |  | 19 | 25 | 17 | <50 & >40 | 1 | 4.0 |
|  |  | 20 | 83 | 451 | <5 & >1 | 17 | 20.5 |
|  | 2 | 1 | 4 | - | <50 & >40 | 1 | 25.0 |
|  |  | 2 | 59 | 24 | <25 & >20 | 7 | 11.9 |
|  |  | 3 | 39 | 140 | <50 & >40 | 10 | 25.6 |
|  |  | 4 | 4 | 141 | <70 & >60 | 4 | 100.0 |
|  |  | 5 | 41 | 49 | <40 & <30 | 2 | 4.9 |
|  |  | 6 | 70 | 64 | <40 & <30 | 16 | 22.9 |
|  |  | 7 | 51 | 85 | <50 & >40 | 1 | 2.0 |
|  |  | 8 | 50 | 15 | <25 & >20 | 3 | 6.0 |
|  |  | 9 | 79 | 87 | <50 & >40 | 17 | 21.5 |
|  |  | 10 | 13 | 12 | <70 & >60 | 13 | 100 |
|  |  | 11 | 38 | 4 | <50 & >40 | 1 | 2.6 |
|  |  | 12 | 51 | 178 | <1 | 16 | 31.4 |
|  |  | 13 | 22 | 174 | <60 & >50 | 21 | 95.5 |
|  |  | 14 | 73 | 23 | <30 & >25 | 2 | 2.7 |
|  |  | 15 | 6 | 281 | <70 & >60 | 6 | 100 |
|  |  | 16 | 37 | 41 | <15 & >10 | 4 | 10.8 |
|  |  | 17 | 12 | 27 | <70 & >60 | 12 | 100 |
|  |  | 18 | 32 | 122 | <60 & >50 | 24 | 75.0 |
|  |  | 19 | 14 | 7 | <60 & >50 | 7 | 50.0 |
|  |  | 20 | 18 | 41 | <60 & >50 | 9 | 50.0 |
|  |  | 21 | 8 | 17 | <60 & >50 | 8 | 100 |
|  |  | 22 | 38 | 324 | <60 & >50 | 25 | 65.8 |
|  |  | 23 | 18 | 129 | <50 & >40 | 6 | 33.3 |
|  |  | 24 | 48 | 293 | <30 & >25 | 6 | 12.5 |
|  |  | 25 | 27 | 7 | <60 & >50 | 17 | 63.0 |
|  |  | 26 | 18 | 633 | <60 & >50 | 2 | 11.1 |
|  |  | 27 | 7 | 120 | <70 & >60 | 7 | 100 |
|  |  | 28 | 160 | 173 | <1 | 109 | 68.1 |
| *H. panicea* | 1 | 1 | 290 | - | < 1 | 225 | 77.6 |
|  |  | 2 | 315 | 585 | < 1 | 135 | 42.9 |
|  |  | 3 | 440 | 505 | < 1 | 180 | 40.9 |
|  |  | 4 | 470 | 1050 | < 1 | 130 | 27.7 |
|  |  | 5 | 160 | 1447 | < 10 & > 5 | 18 | 11.3 |
|  | 2 | 1 | 260 | - | < 1 | 10 | 3.8 |
|  |  | 2 | 660 | 110 | < 1 | 185 | 28.0 |
|  |  | 3 | 110 | 955 | < 1 | 85 | 77.3 |
|  |  | 4 | 30 | 540 | < 60 & > 50 | 20 | 66.7 |
|  |  | 5 | 205 | 290 | < 1 | 35 | 17.1 |
|  |  | 6 | 115 | 540 | < 1 | 90 | 78.3 |
|  |  | 7 | 208 | 572 | < 1 | 161 | 77.4 |
|  |  | 8 | 128 | 594 | < 1 | 91 | 71.1 |
|  | 3 | 1 | 115 | - | < 1 | 90 | 78.3 |
|  |  | 2 | 160 | 200 | < 1 | 80 | 50.0 |
|  |  | 3 | 250 | 525 | < 1 | 185 | 74.0 |
|  |  | 4 | 250 | 755 | < 1 | 215 | 86.0 |
|  |  | 5 | 230 | 940 | < 1 | 205 | 89.1 |
|  |  | 6 | 150 | 1152 | < 1 | 111 | 74.0 |
|  | 4 | 1 | 225 | - | < 1 | 135 | 60.0 |
|  |  | 2 | 115 | 95 | < 50 & > 40 | 5 | 4.3 |
|  |  | 3 | 10 | 255 | < 60 & > 50 | 5 | 50.0 |
|  |  | 4 | 145 | 185 | < 1 | 10 | 6.9 |
|  |  | 5 | 210 | 630 | < 1 | 145 | 69.0 |
|  |  | 6 | 20 | 765 | < 60 & > 50 | 15 | 75.0 |
|  |  | 7 | 15 | 35 | < 50 & > 40 | 5 | 33.3 |
|  |  | 8 | 125 | 20 | < 50 & > 40 | 5 | 4.0 |
|  |  | 9 | 345 | 115 | < 1 | 270 | 78.3 |
|  |  | 10 | 5 | 1264 | < 60 & > 50 | 3 | 60.0 |
|  |  | 11 | 157 | 69 | < 1 | 82 | 52.2 |

**Figure S1.** Temporal dynamics of internal oxygenation in single-osculum demosponge explants. Stacked area plots display the cumulative relative area of the explant base experiencing different oxygen saturation ranges over time for (**A**) *H. rosea* (*ID*1) and (**B**) *H. panicea* (*ID*1, *ID*2, *ID*4) sponges. Oxygen levels (% air saturation) are colour-coded according to the accompanying scale, illustrating transient deoxygenation events in the sponge interior.

**Figure S2.** Spatial distribution and frequency of internal hypoxia and anoxia in single-osculum demosponge explants. The spatial extent of deoxygenation is shown for (**A**) *H. rosea* (*ID*1) and (**B**) *H. panicea* (*ID*1, *ID*2, *ID*4) sponges. **Left column:** Coloured regions delineate the cumulative relative area of the explant base that experienced specific oxygen depletion thresholds (< 10% [green], < 4% [red], and < 1% [blue] air saturation) at least once during the respective observation period. Pixels outside the sponge base are masked in black. **Right column**: Grayscale maps illustrate the relative frequency of anoxia (< 1% air saturation) in each pixel. Darker shades indicate a higher frequency of anoxic events. The darkest pixel (solid black) represents the maximum anoxia frequency, occurring in 0 of 4241 measurements for *H. rosea* (*ID*1) and 111 (*ID*1), 277 (*ID*2) and 155 (*ID*4) of 5526 measurements for *H. panicea*.

**Figure S3.** Spatiotemporal patterns of internal deoxygenation in single-osculum demosponge explants. Space-time plots show oxygen saturation measured in pixels along a vertical transect bisecting the base of (**A**) *H. rosea* (*ID*1) and (**B**) *H. panicea* (*ID*1, *ID*2, *ID*4) individuals. The x-axis denotes time, while the y-axis represents the spatial position along the transect (max. resolution: ~100 μm/pixel). Internal oxygen saturation is encoded using a grayscale gradient of 256 gray levels, ranging from fully oxygenated (white) to anoxic (black).

**Videos S1-S3.** Time-lapse imaging of dynamic spatiotemporal oxygen patterns in *H. rosea* (*ID*2, Figure 5A). (**Video S1**) Regular rotating spiral wave of low oxygen propagating in an anti-clockwise direction (event #11). (**Video S2**) Spiral pattern that dissipates and transitions into a synchronized deoxygenation pattern (events #15 - #16). (**Video S3**) Distinct advancing front of oxygen depletion resembling a unidirectional wave propagating across the sponge base (event #5). Time indicates the duration of each event (i.e. relative time). Colour scale for oxygen levels is provided in Figure 2.
